# Psychometric Properties and Configural Invariance of the Polish – Language Version of the 20-Item Toronto Alexithymia Scale in Non-clinical and Alcohol Addict Persons

**DOI:** 10.3389/fpsyg.2020.01241

**Published:** 2020-06-17

**Authors:** Dawid Konrad Ścigała, Elżbieta Zdankiewicz-Ścigała, Sylwia Bedyńska, Andrzej Kokoszka

**Affiliations:** ^1^Institute of Psychology, The Maria Grzegorzewska University, Warsaw, Poland; ^2^Faculty of Psychology, SWPS University of Social Sciences and Humanities, Warsaw, Poland; ^3^II Department of Psychiatry, Medical University of Warsaw, Warsaw, Poland

**Keywords:** alexithymia, configural invariance, Toronto Alexithymia Scale, TAS-20PL, psychometric factor structure invariance, addiction

## Abstract

**Aim:**

The development and assessment of the psychometric properties of the Polish-language version of the Toronto Alexithymia Scale (Bagby et al., 1994a, b) is described in this article. The aim of this study was to translate the TAS – 20 into Polish and establish the psychometric properties of this instrument evaluating alexithymia.

**Materials and Methods:**

Data were collected via self-report measures from a total sample of 676 participants: a total of 180 participants (115 males and 65 females) diagnosed with alcohol dependence, and 496 control group (347 males and 149 females).

**Results:**

Confirmatory factor analyses found the factor structure of the original English-language TAS 20 for the three subscales translated into Polish: Difficulty in Identifying Feeling (DIF); Difficulty in Describing Feeling (DDF); Externally Oriented Thinking (EOT). All three subscales showed good internal consistency in non-clinical group and two subscales, DIF and DDF in alcohol addict group. Several EOT items loaded poorly on their intended factor.

**Conclusion:**

The results from the present study indicate that the Polish version of the TAS - 20 is a reliable and valid measure of alexithymia with good levels of internal consistency, homogeneity, and construct validity. We conclude that the TAS-20 has, for the most part, adequate psychometric properties, though interpretation should focus only on the total scale score and DIF and DDF subscales, especially in clinical groups.

## Introduction

Alexithymia (coined from the Greek, a = lack, lexis = words, thymos = feeling) is a trait involving difficulties in the cognitive processing of emotions (Nemiah and Sifneos, 1970). The syndrome of alexithymia, which means, according to the idea of this term’s creators, i.e., Nemiah and Sifneos (1970), “the absence of words for emotions,” appeared in psychology literature mainly with reference to patients who suffer from psychosomatic disorders, patients addicted to alcohol and drugs, patients with Posttraumatic Stress Disorder (PTSD), and sociopaths (Sifneos, 1991). Chronic disorders of emotional processes were observed in those patients, however, in symptomatology, qualitatively different from neurotic disorders. The disorders involve difficulties in recognizing, processing, and regulating emotions. Sifneos describes alexithymia as follows: “These deficits involved an inability to identify and use language to describe feelings, inability to differentiate between emotions with their bodily sensations and feelings, paucity of dreams and fantasy life, and a tendency to describe endless details surrounding a particular emotion-arousing episode which was referred to as an operational way of thinking or as ‘pensee operatoire’” (Sifneos, 1991, p. 118). Mattila et al. (2007) stated that alexithymia rates in the general population have been reported to be 9–17% for men and 5–10% for women whereas estimates are as high as 70% in some clinical groups (Bourke et al., 1992). In an overview study, Taylor et al. (1991) state that alexithymia is a disorder of gaining access to one’s own emotional processes in three areas: (a) in the area of mental representation of emotions; (b) in the scope of behavioral indicators; and (c) in the field of physiological indicators. Difficulties which occur in people affected by disorders in cognitive development of feelings can be described metaphorically as “psychological or emotional blindness” to information about emotions experienced by the subject in an intrapsychic and interpersonal context. Weiskrantz (1993) used the term “blindsight” to describe a disorder in which people do not see objects in the part of their field of view, but at the same time they are able to go round these objects when they are on their way. Similarly, it may be concluded that emotions experienced by individuals with a high level of alexithymia constitute “invisible objects” which, with cognitive measures such as avoiding identification and verbalization, try to invalidate and suppress, or focus on the external aspects of purely perceptual phenomena, thus diverting attention from the emotions’ evoking source. Descriptive characteristics related to these cognitive deficits have enabled the isolation of three related factors: difficulty identifying emotions (difficulty identifying feelings, DIF); difficulty describing emotions (difficulty describing feelings, DDF); and operational thinking style (externally orientated thinking style, EOT) (Bagby et al., 1994a, b). Earlier findings of the researchers (Taylor et al., 1990b) regarding deficits which are typical for the syndrome of alexithymia also included a dimension related to limitations in using imagination. This factor was also incorporated in the original version of the questionnaire to investigate alexithymia, i.e., Toronto Alexithymia Scale (TAS-26) (Taylor et al., 1990a). This questionnaire was subject to many adaptations (Fortes et al., 2017), including the adaptation to the Polish conditions (Maruszewski and Ścigała, 1997; Maruszewki and Ścigała, 1998). Further works carried out by a team of Canadian researchers to improve the psychometric properties of the alexithymia test tool resulted in a 20-question version: TAS-20 (Taylor et al., 1990b; Bagby et al., 1994a, b). The reason for such final version was to develop a tool with satisfactory psychometric properties, and the fact that it was assumed that the “Externally Oriented Thinking” factor includes content referring to the “poverty of imaginary life” factor; therefore, there is no need to isolate this dimension separately (Parker et al., 2003). Slightly different results were achieved in the Polish adaptation of the TAS-26 scale (Maruszewski and Ścigała, 1997) where, as a result of exploratory and confirmatory analyses, the following three-factor solution was obtained: difficulties in recognizing feelings and physical sensations; externally oriented thinking, and difficulties using imagination. The studies were carried out on the general population and clinical groups, mainly with psychosomatic disorders (after myocardial infarction and appetite disorders). The results for individual TAS-26 subscales in terms of diagnostic relevance turned out to be interesting. It was found that between the group of healthy individuals and the persons with psychosomatic disorders the highest differences refer to: difficulties in recognizing and differentiating feelings and physical sensations, as well as difficulties in using imagination. On the other hand, the value of the externally oriented thinking factor was nearly identical in the study groups (Maruszewski and Ścigała, 1997). Poverty of imaginary life limits the number of ideas that individuals with high levels of alexithymia can generate in a problem situation. Ideas, which they formulate, are sometimes described as “hyperlogical.” The sources of such ideas are also limited. As a rule, they use external sources, and therefore when dealing with certain problems, they analyze in detail what others have done, and the solutions they create are in numerous cases the modifications of proposals originally formulated by others. Taylor et al. (1990b) found in their studies specific content related changes in the imaginary life of individuals diagnosed with alexithymia. It was stated that those persons’ difficulties relate exclusively to dreams about positively marked events and matters. On the other hand, ideas related to the feeling of guilt and fear of failure appear very easily in them. The fact that this factor was not reflected in the last version of the scale may be considered a limitation when it comes to the search for a theoretical foundation relating to the origin of alexithymia (Maruszewki and Ścigała, 1998; Zdankiewicz-Ścigała, 2017). However, due to the fact that the authors of alexithymia questionnaire developed a new version, i.e., TAS -20, we abandoned further work on the TAS-26 version in Poland. The new version of the scale has been widely applied in studies conducted worldwide, where TAS-20 is the most commonly used tool for diagnosing alexithymia. In numerous countries and cultures, the scale has been adapted to the needs of a given language (Taylor et al., 2003). A thorough verification of the scale’s psychometric properties reveals an important phenomenon, namely that alexithymia may be considered a universal intercultural phenomenon of psychological aspects of individuals’ functioning. In addition, the detection in all analyzed populations of the three-factor nature of alexithymia suggests the existence of common latent personality traits which explain specific disorders in the area of recognition, understanding and regulation of emotions. Specifically, in the work of Taylor et al. (2003) the results of analyses of 18 language versions were discussed, including the Polish version of the scale translated by M. Dąbkowski and J. Rybakowski. The study covered 286 students, i.e., a non-clinical population. Results from CFA revealed that the three-factor structure provided an acceptable fit (χ2/df ratio, 2.32; Goodness-of-Fit Index [GFI], 0.89; Adjusted Goodness of Fit Index [AGFI], 0.86; Standardized Root Mean Square Residual [SRMR], 0.08) (Taylor et al., 2003). Regrettably, no separate publications of these authors appeared regarding the validation of TAS-20. As already mentioned, despite the large number of international studies on psychometric properties of the alexithymia scale, apart from the mentioned unpublished studies in the Polish language, at the moment, there is no widely available study verifying the accuracy of the three-factor scale to investigate alexithymia in the Polish language. In scientific studies, we use the tool, which we initially prepared in 2015 (Zdankiewicz-Ścigała and Ścigała, Unpublished), or the older version of the TAS-26 scale (Zdankiewicz-Ścigała and Strzeszkowska, 2018). Due to the fact that at the moment we have a very large pool of results obtained from various research projects, where we used the TAS-20 scale, we have decided that this is a large enough sample to verify the psychometric properties of the Polish scale for investigating alexithymia.

From the very beginning of the syndrome, intensive research has been underway to verify the role of those deficits in the etiopathogenesis of various disorders; typical psychosomatic disorders (Porcelli and Taylor, 2018); anxiety disorders and depression (Honkalakampi et al., 2000); addictions (Morie and Ridout, 2018; Zdankiewicz-Ścigała and Ścigała, 2018); PTSD (Frewen et al., 2008); Autism Spectrum Disorders (ASD) (Brewer et al., 2015; Gaigg et al., 2016; Poquérusse et al., 2018). The fact of such wide interest in the role of alexithymia in the development and maintenance of non-adaptive mechanisms of broadly understood emotion regulation must direct the scholars’ attention toward the development of a very precise tool which would enable relating the results obtained in a given cultural and linguistic environment to those existing in the world literature. To recapitulate, the purpose of the presented study is to verify the factor structure of the scale, to examine its internal accuracy and reliability on a group of individuals from the clinical population (individuals addicted to alcohol) and on a non-clinical population.

## Materials and Methods

### Participants and Procedure

#### Procedure

All procedures in this study followed (a) the principles of Helsinki Declaration (World Medical Association, 2013), (b) the APA ethical standards (Including 2010 and 2016 Amendments), and (c) the SWPS University of Social Sciences and Humanities Ethics Committee’s guidelines and national regulations. Before starting to fill in the questionnaires, participants were asked to sign an informed consent form which specified all their tasks and rights.

#### Participants

##### Clinical group

The study was carried out among 180 patients of four addiction treatment centers in Warsaw from the 8-week abstinence-based inpatient treatment program combined intensive group and individual therapy as well as elements of 12-step facilitation and relapse prevention. These patients had been diagnosed using the ICD-10 and the MAST questionnaire for diagnosing alcohol addiction. The participants were divided into a control group (a score below 4 points), a group of likely addicted individuals (a score of 4 points), and a group of addicted individuals (a score over 5 points). The study was conducted at the end of the detoxification processes of alcohol-dependent inpatients, including 65 women (36% of participants) and 115 men (64% of participants). The average age was *M* = 41.18; *SD* = 12.99. The TAS-20 results derived from previous research on psychological determinants of alcohol addiction (Zdankiewicz-Ścigała, 2017; Zdankiewicz-Ścigała and Ścigała, 2018). All patients were of Polish nationality, and they were all fluent in the Polish language. The research was carried out in the years 2015 – 2017.

##### Non-clinical group

The control group comprised 496 persons: 149 (30%) women and 347 (70%) men; the average age was *M* = 39.30; *SD* = 12.01. All study participants had at least secondary education, were of Polish nationality, and their native language was Polish. The results of individuals from the control group come from the research previously carried out as part of other study projects (Zdankiewicz-Ścigała, 2017; Zdankiewicz-Ścigała and Ścigała, 2018). The study was carried out in the years 2015 – 2017.

### Materials

The Toronto Alexithymia Scale (TAS-20) (Bagby et al., 1994a) is a 20-item self-report instrument with each item rated on a 5-point Likert scale ranging from 1 (strongly agree) to 5 (strongly disagree); 5 of the items are negatively keyed (4,5,10,18,19). Total scores range between 20 and 100, with higher scores indicating higher degrees of alexithymia, while a person is considered alexithymic with a score equal to or greater than 61. The English original version of the TAS-20 was translated into Polish following the international rules suggested by Brislin (1970) the international guidelines (Hambleton et al., 2005; International Test Commission, 2017). This Polish version of TAS-20-PL like the English version includes 20 self-report questions distributed into three subscales: (1) DIF difficulty identifying feelings and distinguishing between feelings and bodily sensations in emotional activation, (seven items e.g., “I have feelings that I can’t quite identify”), (2) DDF difficulty describing feelings, (five items, e.g., “People tell me to describe my feelings more”), and (3) EOT externally oriented thinking, (eight items, “I prefer to analyze problems rather than just describe them”).

### Analysis

**FIGURE 1:**
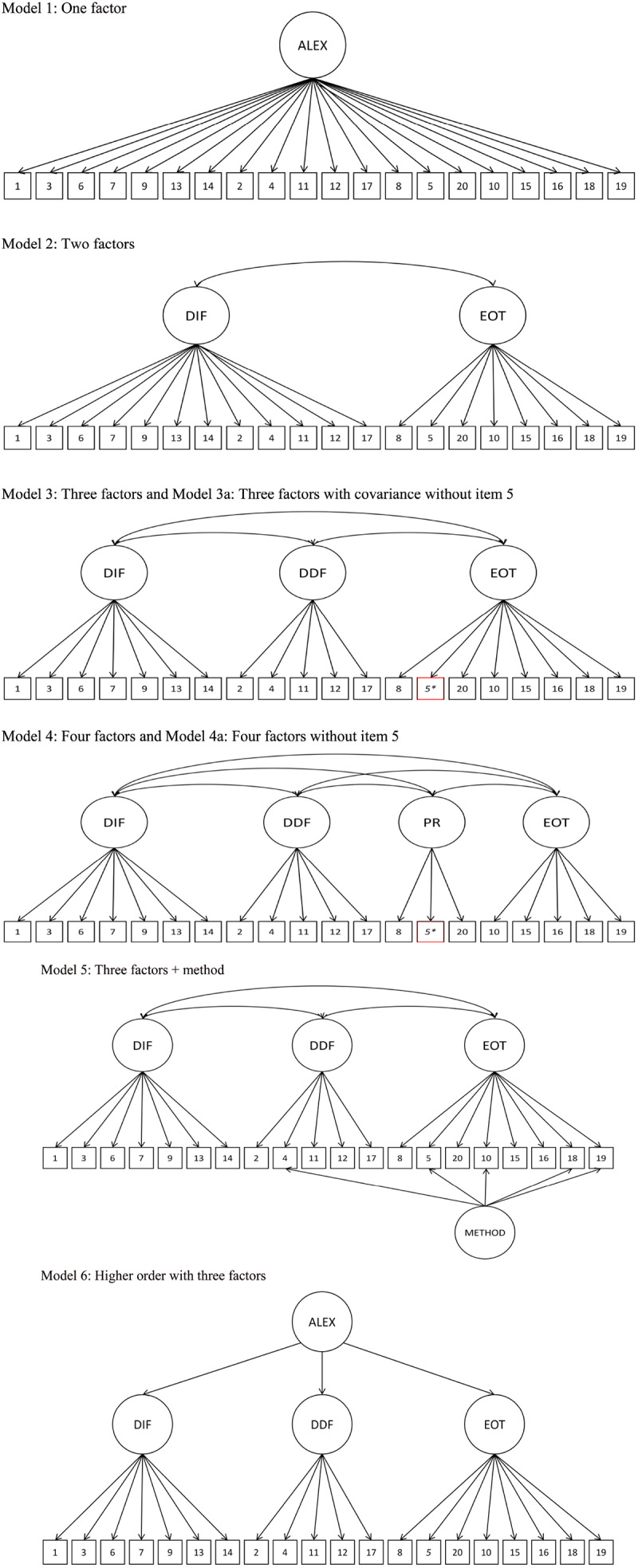
The assessed confirmatory factor analysis models for the TAS-20. Item error terms are not displayed. Alexi, alexithymia; DIF, difficulty identifying feelings; DDF, difficulty describing feelings; EOT, externally orientated thinking; PR, pragmatic thinking; IM, lack of importance of emotions; method, reverse scored item method factor.

Bagby et al. (1994a) were the first to publish the exploratory factor analysis and CFA study which examined the factor structure of the TAS–20. They have found the support for the three-factor model of alexithymia. The CFA fit indexes and evaluation criteria which Bagby et al. (1994a) used to evaluate their models included the goodness-of-fit index (GFI; >85), adjusted-goodness-of-fit index (AGFI; >0.80), and root mean square residual (RMSR; <0.10). In their CFA investigation, Bagby et al. obtained values of GFI = 0.886 and AGFI = 0.856, and accepted the oblique three factor model as satisfactory based on their criteria demarcating good fit. Based on the values reported in Taylor et al. (2003) review of the CFA research on the TAS–20, only 3 out of 24 studies were reported to be associated with a GFI > 0.94. Corresponding with previous research we specified five factor structures (see Figure 1 and models): a unidimensional model (M1: one factor model; model 1), a two dimensional model with DDF and DIF as one factor (M2: two factors; model 2) a three dimensional model with DDF, DIF, and EOT (M3: three correlated factors; model 3), a four factor model with DDF, DIF, PR, and IM (M4: four factors; model 4). Following Preece et al. (2017), we also examined the influence of reverse-scored items on a factor structure using model with method factor (M5; model 5) that is assumed to load all reversed items. We also tested a higher order model with general alexithymia factor for the first-order model (M6; model 6). To evaluate model fit, we used general fit statistics such as the chi square. The confirmatory factor analyses (CFAs) were conducted using AMOS 24, all other analyses used SPSS 25. Initially, descriptive statistics were calculated to explore the requirements of statistical tests. This exploratory analysis were based on means, standard deviations and statistics describing the shape of the distribution such as skewness and kurtosis. The confirmatory factor analysis (CFA) was performed in Mplus 8.2 using Weighted Least Squares Mean and Variance Adjusted (WLSMV) method of estimation. The WLMSV is a robust estimator that does not require normally distributed data (Brown, 2006). Given its sensitivity to sample size, we also considered more specific measures of the model fit such as root mean square error approximation (RMSEA), the Comparative Fit Index (CFI), and the Tucker-Lewis Index (TLI). We used widely recommended cut-off values indicative of adequate model fit to the data, respectively: RMSEA less than 0.08, CFI and TLI above 0.90 (Bentler and Bonett, 1980; Schermelleh-Engel et al., 2003).

## Results

Table 1 reports descriptive statistics, and intergroup comparisons. Based on the data presented in Table 1, it may be concluded that there are statistically significant differences in the level of alexithymia between the clinical group and the control group, both in relation to the average results for the entire scale and the results in individual subscales.

**TABLE 1:** Descriptive statistics and intergroup comparisons for the TAS-20 in the non-clinical and clinical (alcoholic) groups.

	**Non-clinical sample *n* = 496**				**Alcoholic sample *n* = 180**				***F***	***p***	**η2**
	***M***	***SD***	**95%CI^2^**	**95%CI^1^**	***M***	***SD***	**95%CI^1^**	**95%CI^1^**			
Total score	43.93	12.48	42.85	44.99	54.97	12.54	52.97	57.12	*89.082*	*<0.0001*	*0.123*
DIF	15.29	5.86	14.76	15.88	20.17	6.10	19.26	21.16	*83.990*	*<0.0001*	*0.113*
DDF	11.88	4.39	11.49	12.29	15.93	4.43	15.27	16.62	*105.187*	*<0.0001*	*0.138*
EOT	16.86	4.78	16.47	17.24	19.23	4.76	18.53	19.92	*29.687*	*<0.0001*	*0.044*

*^1^Mean (SD), ANOVA, η^2^, and ^2^(BCa) Bootstrap confidence interval are listed.*

Significantly higher results were found in the clinical group of alcohol addicts. Based on the results obtained, a conclusion may be drawn about the existence of criteria, similar to those presented by the authors of the scale (Bagby et al., 1994a, b), for distinguishing the level from which alexithymia may be diagnosed. According to Bagby et al. (1994a, b), the TAS-20 uses cutoff scoring: equal to or less than 51 = non-alexithymia, equal to or greater than 61 = alexithymia. Scores from 52 to 60 = possible alexithymia. While in clinical diagnosis, alexithymia diagnosis according to the scale to: no alexithymia, possible alexithymia, and alexithymia may be extremely useful, in scientific analyses, the division by criterion may limit statistical analyses, and hence a more dimensional approach to examining the level of alexithymia is more useful.

### Internal Consistency Reliability

Table 2 reports correlations for items of Toronto Alexithymia Scale (TAS-20) for clinical sample and for non-clinical sample and Cronbach’s alpha reliability coefficients for the TAS-20 in the clinical and non-clinical samples. Internal consistency of TAS-20 in both samples was calculated using Cronbach’s alpha for all items of the scale and for subscales. As it was reported in the literature (Bagby et al., 2020), the reliability of the EOT subscale was substantially lower than the rest of the subscales (see Table 2).

**TABLE 2:** Pearson correlations and Cronbach’s alpha reliability coefficients for the TAS-20 in the non-clinical and clinical (alcoholic) groups.

	**Non-clinical sample *n* = 496**					**Alcoholic sample *n* = 180**				
	**α**	**Total**	**DIF**	**DDF**	**EOT**	**α**	**Total**	**DIF**	**DDF**	**EOT**
Total score	0.86	–				0.82	–			
DIF	0.81	0.865**	–			0.74	0.881**	–		
DDF	0.75	0.861**	0.674**	–		0.74	0.816**	0.631**	–	
EOT	0.64	0.752**	0.410**	0.499**	–	0.51	0.721**	0.425**	0.381**	–

DIF, difficulty identifying feelings; DDF, difficulty describing feelings, EOT, externally oriented thinking; α, Cronbach’s alpha. ** *p* < 0.001.

The analysis of results presented in Table 2 allows to state that the reliability coefficient for the whole scale and subscales is higher in the control group than in the clinical group, although it is also satisfactory in this group. Noteworthy is the analysis of values obtained for the EOT scale, whose reliability differs significantly from the reliability of the other scales. It is also worth noting that it is lower in the clinical group (see Table 2). It is important, however, that in both the control and clinical groups, the results of this scale correlate satisfactorily with the results of the whole scale for examining alexithymia. The internal consistency of the DIF and DDF was at the acceptable level considering small sample size in the clinical sample and relatively small number of items in the subscales. The results obtained in this study are very consistent with the results obtained by other authors (see Table 3).

**TABLE 3:** Psychometric properties of the TAS-20 in control and a similar clinical group in us and other researchers.

**Authors**	**Samples**	**Sex**	**Age**	**CFA**	**Control**	**Clinical**
					**α**	**α**
Ścigała et al., 2020	Control *n* = 596	F-30%; M-70%	*M* = 39.30; *SD* = 12.01	TAS	0.86	0.82
	Substance addictive *n* = 180	F-36%; M-64%	*M* = 41.18; *SD* = 12.99	DIF	0.81	0.74
				DDF	0.75	0.74
				EOT	0.64	0.51
Bagby et al., 1994a	Control *n* = 401	F-60%; M-40%	*M* = 21.1; *SD* = 4.2	TAS	0.80	0.83
	Clinical *n* = 218	F-57%; M-43%	*M* = 35.2; *SD* = 11.5	DIF	0.79	0.81
	15.6% Anxiety			DDF	0.75	0.75
	15.6% Somatoform			EOT	0.66	0.64
	11.1% Personality disorder					
	10.6% Dysthymia					
	10.1% Major depression					
Loas et al., 2001	Control *n* = 769	F-60%; M-40%	*M* = 27.09; *SD* = 8.58	TAS	0.78	0.74
	Substance addictive and	F-59%; M-41%	*M* = 27.18; *SD* = 8.64	DIF	0.74	0.73
	Eating disorder *n* = 659			DDF	0.71	0.61
				EOT	0.56	0.56
Meganck et al., 2008	Control *n* = 157	F-85%; M-15%	*M* = 20.73; *SD* = 2.53	TAS	0.78	0.80
	Clinical *n* = 404	F-70%; M-30%	*M* = 38.4; *SD* = 10.6	DIF	0.81	0.82
	44% Mood disorder			DDF	0.70	0.78
	15% Anxiety			EOT	0.53	0.56
	4% Adjustment					
	3% Substance addictive					
	2% Eating disorder					
	11% Other clinical					
Preece et al., 2017	Control *n* = 428	F-61%; M-39%	*M* = 41.62; *SD* = 16.77	TAS	87	86
	Clinical *n* = 156	F-71%; M-29%	*M* = 41.10; *SD* = 12.17	DIF	87	87
	49.4% Mood disorder			DDF	82	74
	26.9% Neurotic and stress related			EOT	64	61
	14.1% Personality disorders					
	6.4% Schizophrenia					

*CFA, Confirmatory Factor Analysis- three factor model. TAS, Alexithymia total; DIF, difficulty identifying feelings; DDF, difficulty describing feelings; EOT, externally oriented.*

It has been indicated through comparing them with the results of 25 years of studies on psychometric properties of the alexithymia scale which have been summarized in the publication that has just been released (Bagby et al., 2020). The most controversial aspects, as may be concluded from the article, which were also observed by the authors, as well as discussed by the aforementioned authors, relate to the reliability of the EOT scale. However, considering the results of psychometric studies conducted in many other countries and cultures on a large group of subjects, both clinical and non-clinical, the authors suggest that the scale has adequate-to-excellent internal reliability and the lower alpha coefficients for the EOT factor scale have been suggested in the literature, including cultural differences and / or a response bias to the reverse scored items on this factor scale (Bagby et al., 2020).

### Confirmatory Factor Analysis

Model fit statistics for all five models tested in CFA are presented in Table 4.

**TABLE 4:** Model fit statistics in confirmatory Factor Analysis for clinical and non-clinical samples.

	**χ^2^ (df)**	**TLI**	**CFI**	**RMSEA (90% CI)**
**Clinical sample**				
M1: one factor	536.28 (170)	0.73	0.76	0.112 [0.101, 0.122]
M2: two factors	1018.22 (169)	0.86	0.87	0.101 [0.095, 0.107]
M3: three factors	506.49 (167)	0.75	0.78	0.108 [0.098, 0.119]
M3a: three factors with covariance without item 5	902.445 (168)	0.88	0.89	0.094 [0.088, 0.100]
M4: four factors	500.579 (164)	0.75	0.78	0.109 [0.098, 0.120]
M4a: four factors without item 5	479.441 (165)	0.77	0.80	0.105 [0.094, 0.116]
M5: three factors + method	405.647 (159)	0.81	0.84	0.095 [0.083, 0.106]
M6: higher order with three factors	506.49 (167)	0.75	0.78	0.108 [0.098, 0.119]
**Non-clinical sample**				
M1: one factor	1426.80 (170)	0.79	0.81	0.122 [0.116, 0.128]
M2: two factors	1018.22 (169)	0.86	0.87	0.101 [0.095, 0.107]
M3: three factors	955.06 (167)	0.87	0.88	0.098 [0.092, 0.104]
M3a: three factors with covariance	678.08 (164)	0.91	0.92	0.079 [0.073, 0.086]
M4: four factors	961.27 (164)	0.86	0.88	0.099 [0.093, 0.105]
M5: three factors + method	No convergence achieved with 100,000 iterations			
M6: higher order with three factors	955.06 (167)	0.87	0.88	0.098 [0.092, 0.104]

*All χ^*2*^< 0.001, RMSEA, Root Mean Square Error Approximation; CFI, Comparative Fit Index; TLI, Tucker-Lewis Index; CI, Confidence intervals.*

The series of analyses, which was performed first, concerned the study of basic models that may most accurately reflect the structure of TAS-20 (see Figure 1). In the test, the first-order factor structures were as follows: M1: a single-factor model in which all questions were included in one factor; M2: a two-factor model in which two DIF and DDF and EOT factors are combined into one factor; M3 the traditional three-factor correlated model where items were specified to load on a DIF, DDF, or EOT factor; and M3a traditional three-factor correlated model where items were specified to load on a DIF, DDF, or EOT factor with covariance without item 5. The goodness-of- fit models were judged based on the pattern of factor loadings and intercorrelations within each model, and three fit indices: *X*^2^, Tucker-Lewis index (TLI), the comparative fit index (CFI), and root mean square error of approximation (RMSEA). CFI and TLI values ≥0.90 were judged to indicate acceptable fit, as were RMSEA values ≤0.08 (Bentler and Bonett, 1980). These three fit indices were selected as they are considered to be among the best indicators of model fit (Byrne, 2016). CFA indicated that among all first-order models, the model with three correlated factors achieved the best fit. In the clinical sample this model was modified as item 5 was not correlated to EOT factor. In subsequent stages, the models were analyzed with the addition of a method factor loading on the reverse-scored items in both clinical and control groups (Figure 1, model 5). The three-factor correlated model + method demonstrated acceptable levels of the fit only in the clinical sample according to RMSEA, though fit was still unacceptable according to CFI and TLI. On the basis of modification indices, we also added covariance between error terms of item 3 and item 7. Similar covariance item 3 and item 7 error terms was also entered to the model in Preece and colleagues work (Preece et al., 2017) and it improved fit of the model. The three-factor correlated model + method + covariance, however, still did not quite reach globally acceptable levels of fit. In the non-clinical sample there was no need to reduce this model because item 5 was significantly related to the EOT factor. The size of the factor score for item 5 was lower than 0.40 showing weak correlation with EOT. Again inspection of the modification indices suggested to allow covariance between error terms of item 3 and item 7. However, none of the model achieved acceptable level of fit in all statistics in the clinical sample. In the non-clinical sample only three factor model obtained generally acceptable model fit. Factor loadings for all models for Toronto Alexithymia Scale in both groups are presented in Table 5.

**TABLE 5:** Factor scores and standard errors in the 3-factors model, 3-factors + method model, 4 – factors model in the clinical and non-clinical samples.

**Factor**		**Item**	**Clinical sample**			**Non-clinical sample**		
			**M3a: 3-factor without item 5**	**M5: 3-factor + method**	**M4: 4-factor**	**M3a: 3-factor without item 5**	**M5: 3-factor + method**	**M4: 4-factor**
DIF		1	0.768 (0.026)	0.603 (0.055)	0.601 (0.055)	0.767 (0.026)	No convergence	0.767 (0.026)
		3	0.453 (0.042)	0.540 (0.061)	0.546 (0.061)	0.454 (0.042)		0.455 (0.042)
		6	0.697 (0.027)	0.694 (0.043)	0.692 (0.044)	0.697 (0.027)		0.697 (0.026)
		7	0.612 (0.032)	0.483 (0.065)	0.485 (0.065)	0.613 (0.032)		0.613 (0.032)
		9	0.751 (0.024)	0.737 (0.041)	0.738 (0.041)	0.751 (0.024)		0.750 (0.024)
		13	0.776 (0.024)	0.714 (0.038)	0.717 (0.038)	0.776 (0.024)		0.775 (0.024)
		14	0.740 (0.026)	0.713 (0.041)	0.710 (0.041)	0.740 (0.026)		0.740 (0.026)
DDF		2	0.781 (0.023)	0.741 (0.040)	0.760 (0.038)	0.781 (0.023)		0.781 (0.023)
		4	0.714 (0.026)	4.653 (11.11)	0.594 (.041)	0.716 (0.026)		0.717 (0.026)
		11	0.750 (0.025)	0.767 (0.042)	0.792 (0.041)	0.750 (0.025)		0.750 (0.025)
		12	0.468 (0.037)	0.381 (0.070)	0.392 (0.072)	0.466 (0.037)		0.466 (0.037)
		17	0.633 (0.031)	0.502 (0.061)	0.509 (0.63)	0.629 (0.031)		0.629 (0.031)
EOT	PR	8	0.471 (0.044)	0.481 (0.066)	0.421 (0.099)	0.468 (0.044)		0.440 (0.052)
		20	0.540 (0.041)	0.423 (0.075)	0.377 (0.107)	0.538 (0.041)		0.505 (0.058)
		5	–	–	0.021 (0.068)	0.144 (0.052)		0.149 (0.050)
	IM	10	0.695 (0.040)	2.679 (1.323)	0.252 (0.089)	0.698 (0.040)		0.705 (0.039)
		15	0.637 (0.038)	0.515 (0.066)	0.626 (0.088)	0.629 (0.038)		0.638 (0.040)
		16	0.505 (0.041)	0.308 (0.073)	0.370 (0.090)	0.497 (0.042)		0.504 (0.043)
		18	0.588 (0.040)	1.648 (0.804)	0.400 (0.086)	0.589 (0.040)		0.595 (0.040)
		19	0.771 (0.031)	4.948 (2.813)	0.490 (0.077)	0.768 (0.031)		0.779 (0.030)

*DIF, difficulty identifying feelings; DDF, difficulty describing feelings; EOT, externally oriented thinking; PR, pragmatic thinking; IM, lack of importance of emotions.*

Higher order models and model with method factor, taking into account reversed coded items presented a slightly worse fit in both samples than first-order three factors model. In the non-clinical sample model with method component did not achieve convergence even though the number of iterations was set to 100,000.

## Discussion of the Results

The purpose of the study was to determine the psychometric properties of TAS-20 PL. The obtained results indicate that the Polish adaptation of TAS-20 may constitute a useful tool for assessing the intensity of alexithymia in the Polish population. The factor structure of the original tool was also confirmed in this study. The results obtained in the analyses indicate favorable and comparable to the original psychometric characteristics of the Polish translation. As a result of exploratory and CFA, the fitting of models verified in original studies was tested. The model with three latent variables (20 TAS items), i.e., (1) DIF, (2) DDF, (3) EOT was tested with WLSMV method. The satisfactory model fitting to the data was stated (see Table 2) both for non-clinical and clinical groups. These results fully replicate the data obtained in original studies (Taylor et al., 1990b; Bagby et al., 1992). The factor structure of the scale corresponds to the theoretical conceptualization of the alexithymia construct. Out of the examined first-order correlated models, the three-factor correlated model (DIF, DDF, EOT) is the most optimal solution for both examined groups. Our results indicate that the TAS-20 PL has, in its main part, adequate psychometric properties, although the EOT subscale and questions with inverse points appear problematic. The results obtained are consistent with those of other studies (e.g., Kooiman et al., 2002; Gignac et al., 2007; Meganck et al., 2008; Loas et al., 2017; Preece et al., 2017). One of the reasons quoted in literature regarding the relatively low psychometric properties of the EOT scale is the fact of inverse scores for many questions in this scale. However, the analyses carried out by the abovementioned researchers and by us indicate that removing inversely scored questions from the model did not improve the low internal consistency of this subscale. With respect to the clinical group, a good fit of the three-factor structure of alexithymia was obtained after removing question 5 from the EOT scale. In the case of the non-clinical group, such action was not necessary. Therefore, it is difficult to conclude that the low internal consistency of this subscale results from the translation of questions into Polish and, as the authors of the scale suggest, from a different meaning that respondents may assign to individual questions. As it results from the analysis of the TAS-20 scale adaptation carried out in numerous cultures and countries, summarized by Bagby et al. (2020), problems with certain questions in this subscale are relatively common. The quoted authors claim that “most of these later translations show adequate to good internal reliability for the total scale and the DIF and DDF factor scales; but again, lower estimates of internal reliability are reported for the EOT factor scale in some but not all studies” (Bagby et al., 2020, p. 8). This does not change the fact that in the overall fit, it is the scale consisting of three factors that satisfactorily meets the criteria of psychometric relevance. TAS-20Pl may be used in the studies carried out in Poland. Especially since the overall score of the scale fully differentiates clinical groups from non-clinical groups (e.g., Zdankiewicz-Ścigała and Ścigała, 2018). As suggested by Bagby et al. (2020), when applying the TAS-20 scale for research, it is also worth using other questionnaires that allow the verification of a distortion degree in cognitive processing of emotogenic stimulation. Deficits related to the processing of emotional stimuli refer to distortions: (1) in identifying emotional stimuli (e.g., Mattila et al., 2008; Starita et al., 2018), (2) in recognizing the physiological correlates of stimulus stimulation (e.g., Pollatos et al., 2008), (3) in the regulation of emotional arousal (e.g., Pollatos and Graman, 2012), (4) in the use of information contained in a given emotion in the decision-making process (e.g., Scarpazza et al., 2017). This means that apart from self-report questionnaires for examining alexithymia, it is worth focusing on experimental studies, whose aim to detect the mechanisms underlying these deficits. There is a chance that such studies would provide new data regarding the controversial dimension of EOT. In fact, it refers to cognitive distortions in avoiding introspection. Promising results in this area were obtained from the studies carried out by Starita and di Pellegrino (2018). Numerous recent studies also combine alexithymia with interoception. The ability to interpret interoceptive body signals is necessary to accurately recognize and experience emotions (Brewer et al., 2015; Murphy et al., 2018; Zamariola et al., 2018). Interoceptive deficits in people with alexithymia refer not only to emotions, but also to non-affective signals from the body, which brings the conclusion that alexithymia is a general deficit of interoception (Brewer et al., 2016; Murphy et al., 2018). This may mean that it is worth using questionnaires in the studies to investigate interoception (Mehling et al., 2012). Zamariola et al. (2018) while examining the relationship between alexithymia and interoception, point out that early models relating to alexithymia referred to interoception deficits both at the subjective and objective levels (Taylor et al., 1999).

## Conclusion

As a result of the adaptation procedure, the Polish version of TAS-20 was created. The preliminary works on the Polish adaptation of the scale allowed to determine the psychometric properties of the tool. Further validation works are necessary as they have not been carried out within the presented studies, which resulted in their limitation. Despite that, it may be concluded that the current version of the TAS-20 scale gives the opportunity to use a valuable method in Polish research studies, and therefore deserves to be presented - even at this stage of adaptation works. The usefulness of the discussed research tool results from its following properties: (1) it allows for easy and quick measurement of alexithymia both in categorical approach (absence of alexithymia, possible alexithymia, alexithymia) and in the dimensional approach (three dimensions of alexithymia). This is an important advantage, because – as already noted – recent studies indicate that the dimensional (rather than categorical) model better reflects the nature of alexithymia, and (2) it has satisfactory psychometric properties, corresponding to the properties of the original tool, and its test positions are characterized by the clarity of wording and an accessible format of responses. Based on the presented analyses, TAS-20 may be considered an accurate and reliable tool for measuring deficits in cognitive processing of emotions. The presented studies confirmed the theoretical and criterial accuracy, three-factor structure, and internal consistency of the DIF and DDF scales. Although the results of the analyses indicate that the Polish version is a tool with sufficient internal consistency, further studies with this respect are necessary, especially with regard to the EOT scale. To sum up, the obtained psychometric parameters allow to think that the Polish adaptation of Toronto Alexithymia Scale-20 is a tool with satisfactory psychometric properties, enabling accurate and reliable measurement of the alexithymia phenomenon in both the non-clinical and clinical populations. It may be successfully used in scientific studies on the psychopathology of numerous mental disorders, as well as in cognitive conceptualization, treatment planning, and monitoring the effectiveness of therapy of persons addicted to alcohol, other psychoactive substances or food.

## Data Availability Statement

The datasets generated for this study are available on request to the corresponding author.

## Ethics Statement

All procedures performed in studies involving human participants were in accordance with the ethical standards of the Institutional and/or National Research Committee and with the 1964 Declaration of Helsinki and its later amendments or comparable ethical standards. The study was reviewed and approved by the SWPS University of Social Sciences and Humanities Ethics Committee.

## Author Contributions

EZ-Ś and DŚ made substantial contributions to the conception, design of the work, and acquisition. DŚ and SB analyzed the data. EZ-Ś, DŚ, and SB interpreted the data for the work, drafted the work or revised it critically for important intellectual content. EZ-Ś, DŚ, SB, and AK approved the final version to be published, and agreed to be accountable for all aspects of the work in ensuring that questions related to the accuracy or integrity of any part of the work are appropriately investigated and resolved.

## Conflict of Interest

The authors declare that the research was conducted in the absence of any commercial or financial relationships that could be construed as a potential conflict of interest.
